# Overall Survival Among Patients With De Novo Stage IV Metastatic and Distant Metastatic Recurrent Non–Small Cell Lung Cancer

**DOI:** 10.1001/jamanetworkopen.2023.35813

**Published:** 2023-09-26

**Authors:** Chloe C. Su, Julie T. Wu, Eunji Choi, Nathaniel J. Myall, Joel W. Neal, Allison W. Kurian, Henning Stehr, Douglas Wood, Solomon M. Henry, Leah M. Backhus, Ann N. Leung, Heather A. Wakelee, Summer S. Han

**Affiliations:** 1 Department of Epidemiology and Population Health, Stanford University School of Medicine, Stanford, California; 2 Quantitative Sciences Unit, Department of Medicine, Stanford University School of Medicine, Stanford, California; 3 Division of Oncology, Department of Medicine, Stanford University School of Medicine, Stanford, California; 4 Veterans Affairs Palo Alto Healthcare System, Palo Alto, California; 5 Stanford Cancer Institute, Stanford University School of Medicine, Stanford, California; 6 Research Informatics Center, Department of Medicine, Stanford University School of Medicine, Stanford, California; 7 Department of Cardiothoracic Surgery, Stanford University School of Medicine, Stanford, California; 8 Department of Radiology, Stanford University School of Medicine, Stanford, California; 9 Department of Neurosurgery, Stanford University School of Medicine, Stanford, California

## Abstract

**Importance:**

Despite recent breakthroughs in therapy, advanced lung cancer still poses a therapeutic challenge. The survival profile of patients with metastatic lung cancer remains poorly understood by metastatic disease type (ie, de novo stage IV vs distant recurrence).

**Objective:**

To evaluate the association of metastatic disease type on overall survival (OS) among patients with non–small cell lung cancer (NSCLC) and to identify potential mechanisms underlying any survival difference.

**Design, Setting, and Participants:**

Cohort study of a national US population based at a tertiary referral center in the San Francisco Bay Area using participant data from the National Lung Screening Trial (NLST) who were enrolled between 2002 and 2004 and followed up for up to 7 years as the primary cohort and patient data from Stanford Healthcare (SHC) for diagnoses between 2009 and 2019 and followed up for up to 13 years as the validation cohort. Participants from NLST with de novo metastatic or distant recurrent NSCLC diagnoses were included. Data were analyzed from January 2021 to March 2023.

**Exposures:**

De novo stage IV vs distant recurrent metastatic disease.

**Main Outcomes and Measures:**

OS after diagnosis of metastatic disease.

**Results:**

The NLST and SHC cohort consisted of 660 and 180 participants, respectively (411 men [62.3%] vs 109 men [60.6%], 602 White participants [91.2%] vs 111 White participants [61.7%], and mean [SD] age of 66.8 [5.5] vs 71.4 [7.9] years at metastasis, respectively). Patients with distant recurrence showed significantly better OS than patients with de novo metastasis (adjusted hazard ratio [aHR], 0.72; 95% CI, 0.60-0.87; *P* < .001) in NLST, which was replicated in SHC (aHR, 0.64; 95% CI, 0.43-0.96; *P* = .03). In SHC, patients with de novo metastasis more frequently progressed to the bone (63 patients with de novo metastasis [52.5%] vs 19 patients with distant recurrence [31.7%]) or pleura (40 patients with de novo metastasis [33.3%] vs 8 patients with distant recurrence [13.3%]) than patients with distant recurrence and were primarily detected through symptoms (102 patients [85.0%]) as compared with posttreatment surveillance (47 patients [78.3%]) in the latter. The main finding remained consistent after further adjusting for metastasis sites and detection methods.

**Conclusions and Relevance:**

In this cohort study, patients with distant recurrent NSCLC had significantly better OS than those with de novo disease, and the latter group was associated with characteristics that may affect overall survival. This finding can help inform future clinical trial designs to ensure a balance for baseline patient characteristics.

## Key Points

**Question:**

Is there a survival difference between de novo stage IV and distant recurrent metastatic non–small cell lung cancer (NSCLC)?

**Findings:**

In a cohort study of 660 participants, patients with distant recurrent NSCLC had significantly better survival than those with de novo disease, which was validated in an independent cohort.

**Meaning:**

These findings suggest metastatic disease type as a factor associated with outcomes could be considered in future clinical trial design to ensure a balance for baseline patient characteristics.

## Teaser

This cohort study evaluates whether there is a survival difference between de novo stage IV and distant recurrent metastatic non–small cell lung cancer.

## Introduction

Despite recent breakthroughs in early detection and therapies, advanced-stage lung cancer still poses a therapeutic challenge with high mortality. Nearly half of patients with lung cancer receive a diagnosis at an advanced stage (ie, de novo stage IV),^1^ and one-third of patients with early stage (stages I through III) lung cancer diagnoses will relapse and develop advanced disease (ie, recurrence).^2,3^ It is notable that most major clinical trials for advanced lung cancer have not distinguished between these 2 types of metastatic disease for lung cancer,^4,5,6,7^ even though prior literature has suggested a significantly higher overall survival (OS) among those with recurrence.^8,9^ Such heterogeneity in prognosis may result in a biased estimate of treatment outcomes or limit generalizability if not balanced at baseline in a clinical trial, or if unaccounted for in cost-effectiveness analyses.

Despite prior studies^8,9^ suggesting differential survival by type of metastasis in lung cancer—with better survival observed in patients with recurrence than those with de novo metastasis—this finding is not universal among all cancers. In the breast cancer setting, patients with de novo metastasis are observed to have better survival than patients with recurrence likely related to tumor resistance to prior treatment among patients with recurrence.^10,11,12,13^ By contrast, no survival difference was found in colorectal cancers among these populations.^8^ Thus, the survival differences in metastatic lung cancer are unique and intriguing. However, findings from prior studies in lung cancer have not been fully validated in the US. Although Moore et al^9^ reported better survival for patients with distant recurrence in a population-based cohort in British Columbia, Canada, Hassett et al^8^ suggested that better survival among recurrence may be driven by regional recurrence. Importantly, mechanisms underlying the survival difference between patients with de novo metastasis vs patients with lung cancer recurrence were not investigated.

In this study, we aim to evaluate the survival difference by metastatic disease type—de novo metastasis vs disease recurrence, with a focus on patients with non–small cell lung cancer (NSCLC), using well-curated, US national-level, population-based data from the National Lung Screening Trial (NLST). To ensure a fair comparison, we include only patients with distant recurrence and de novo metastasis in our primary analysis, excluding those with regional recurrence. We evaluate the association between metastatic disease type and OS after diagnosis of distant metastatic disease, adjusting for potential confounders. Furthermore, we validate this finding in the clinical setting and examine the potential drivers of survival difference using data curated from electronic health records (EHR) at Stanford Healthcare (SHC).

## Methods

### Primary Study Cohort

**Figure 1.**
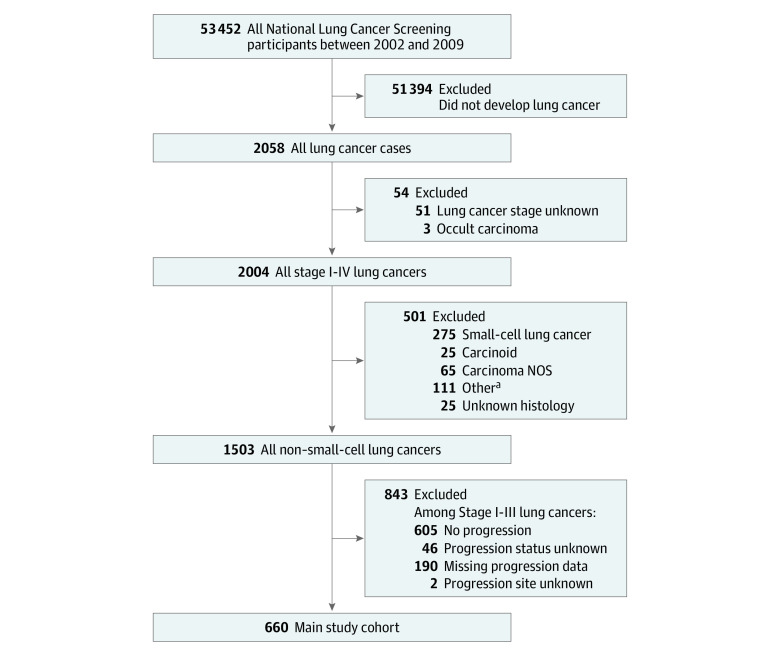
Overview of Study Cohort The National Lung Screening Trial enrolled 52 342 participants, of whom 2058 developed primary lung cancer between 2002 and 2009. Among 2058 cases, 51 cases with unknown lung cancer stage and 3 cases of occult carcinoma were excluded. Of the remaining 2004 stage I to IV lung cancers, a total of 1503 non–small cell lung cancers were identified after excluding 501 other lung cancers. After excluding an additional 843 cases due to no progression, unknown progression, or missing progression data, the main study cohort is made up of 660 cases. NOS indicates not otherwise specified. ^a^Classification of other histologic findings based on *International Classification of Diseases for Oncology, Third Revision* codes including 8000, 8001, 8022, 8032, 8033, 8083, 8084, 8260, 8310, 8323, 8480, 8481, 8490, 8550, 8560, 8570, 8980.

The NLST is a prospective, large, population-based randomized trial that enrolled 53 452 participants between 2002 and 2004 and followed up with them through 2009. The present study included NLST participants who either received diagnoses of de novo stage IV metastatic NSCLC or have documented metastatic disease recurrence after an initial diagnosis of stage I through III NSCLC. Participants with lung cancer types other than NSCLC, who had unknown cancer stage or occult carcinoma, or who had unknown or missing progression status were excluded (Figure 1). Among this study cohort, the primary analysis was conducted with complete case participants who received diagnoses of either de novo metastases or distant recurrence, excluding patients with only regional metastases. This study was reviewed and approved by the Stanford University institutional review board and followed the Strengthening the Reporting of Observational Studies in Epidemiology (STROBE) reporting guidelines. The NLST data were obtained through application from the National Cancer Institute and did not require written informed consent because data were deidentified.

### Validation Cohort

The validation cohort consisted of patients with metastatic NSCLC with molecular testing performed between 2009 and 2019, followed-up through August 2022 in SHC. Medical records of patients, including progress notes and radiology reports, were manually reviewed. Clinical variables, including ECOG performance status, method for detecting metastatic disease, tumor burden, driver variations, treatments received for metastatic disease, and progression sites were curated. The full set of variables curated is listed in eTable 1 in Supplement 1, and details on their definitions and curation methods are included in eMethods 1 in Supplement 1. Of these patients, those with confirmed metastatic disease were included in the validation cohort (eFigure 1 in Supplement 1).

### Outcomes and Primary Variables

The primary outcome was OS after diagnosis of de novo metastasis or distant recurrence. We incorporated data on time from diagnosis of metastases to death or censored (ie, loss to follow-up or alive at the end of the study), whichever occurred first. The primary exposure variable was metastatic disease type (ie, de novo metastasis vs distant recurrence). De novo metastasis was defined as an initial diagnosis of NSCLC at stage IV, while recurrent metastatic disease was defined as diagnosis of metastasis after an initial diagnosis of stage I through III NSCLC. Distant recurrence was defined as progression to pleura, contralateral lung, skin, adrenal, bone, liver, brain, and/or any other distant organs outside the chest after early-stage disease. Regional metastatic disease was defined as progression in the original lung and/or to thoracic lymph nodes.

### Statistical Analysis

We applied multivariable Cox proportional hazards regression to evaluate association between metastatic disease type and OS after diagnosis of metastatic disease. The model was adjusted for potential confounding factors, including age at metastasis, sex, self-reported race, histologic findings of NSCLC, mode of cancer screening (chest radiograph vs low-dose computed tomography [LDCT]) in the NLST cohort, and smoking status at the time of randomization in NLST.^14,15^ Race was assessed as it has been reported to be associated with survival outcomes in lung cancer even in a screening population.^16^ As sensitivity analyses, we restricted the cohort to patients who have received curative therapy (ie, surgery or radiation). Moreover, we conducted an unadjusted Cox proportional hazards regression using propensity score matching (PSM),^17^ with each exposure group of patients matched 1:1 on age, sex, race, marital status, smoking status, histologic findings of NSCLC, screening method, and comorbidity (eMethods 2, eMethods 3, and eTable 2 in Supplement 1). For comparability with prior studies, we repeated the primary analysis, including additional patients with regional recurrence. A 2-sided *P* value of less than .05 was considered statistically significant. All statistical analysis was performed using R version 4.1.2 (R Project for Statistical Computing). Data were analyzed from January 2021 to March 2023.

In the validation cohort from SHC, we replicated the primary analysis but adjusted the model only for age at metastasis, sex, race, and histologic findings of NSCLC. The mode of initial cancer detection was curated but excluded due to rare cases of LDCT screening detection. Smoking status was also excluded due to a lack of timely updates in the EHR. As the missing rate of the covariates was low at 0% to 2.2% (eTable 1 in Supplement 1), we conducted a complete case analysis.

To investigate any survival difference observed, we evaluated the association between metastatic disease type and clinical variables, including driver variations present, sites of metastasis, treatments received for metastatic disease, method of detection for metastatic disease, and tumor burden at the time of metastatic disease (using the 75th percentile of the SHC cohort of 141.75 mm as the cutoff for high tumor burden) (full list in eTable 1 in Supplement 1), using a χ^2^ test. As sensitivity analysis to control for additional potential confounders, we restricted the cohort to only patients who received curative-intent therapy at initial diagnosis for those with distant recurrence and additionally adjusted for any variables showing statistically significant difference by metastatic disease type.

## Results

### Baseline Characteristics of the NLST Cohort

The NLST cohort (660 patients) consisted of 411 male patients (62.3%), 602 White patients (91.2%), 344 patients with adenocarcinoma in histologic findings (52.1%), and 392 current smokers (vs former smoker) at the time of enrollment (59.4%), with a mean (SD) age at the time of metastatic disease of 66.8 (5.5) years. Nearly 60% of the patients had de novo stage IV NSCLC (392 patients), and 40.6% patients had disease recurrence (268 patients) (Table 1). Among the 85.4% of recurrent patients with distant recurrence (229 patients), 64.9% had curative-intent therapy (174 patients). The median (IQR) follow-up time from diagnosis of metastatic disease to death or censoring was 0.5 (0.2-1.0) years for patients with de novo metastases, 0.7 (0.3-1.6) years for those with distant recurrence, and 0.7 (0.3-2.1) years for those with regional recurrence (eFigure 2 in Supplement 1).

**Table 1.** Characteristics of the NLST Study Cohort Grouped by Type of Metastatic Disease

Characteristic	Patients, No. (%)		
	Total (N = 660)	De novo stage IV metastatic disease (n = 392)^a^	Recurrent metastatic disease (n = 268)^a^
Follow-up time, y			
Median (IQR)	0.5 (0.2-1.3)	0.5 (0.2-1.0)	0.7 (0.3-1.7)
Screening information			
Screening method in NLST			
LDCT	301 (45.6)	155 (39.5)	146 (54.5)
Chest radiograph	359 (54.4)	237 (60.5)	122 (45.5)
Demographics			
Age at LC diagnosis, y			
Mean (SD)	66.1 (5.4)	66.5 (5.3)	65.6 (5.6)
Age at diagnosis of metastatic disease, y			
Mean (SD)	66.8 (5.5)	66.5 (5.3)	67.1 (5.7)
Sex			
Female	249 (37.7)	136 (34.7)	113 (42.2)
Male	411 (62.3)	256 (65.3)	155 (57.8)
Race			
Asian	8 (1.2)	5 (1.3)	3 (1.1)
Black	31 (4.7)	17 (4.3)	14 (5.2)
White	602 (91.2)	359 (91.6)	243 (90.7)
Other^b^	12 (1.8)	5 (1.3)	7 (2.6)
Data missing	7 (1.1)	6 (1.5)	1 (0.4)
Marital status			
Married/living together	427 (64.7)	250 (63.8)	177 (66.0)
Not married/not living together^c^	223 (33.8)	133 (33.9)	90 (33.6)
Data missing	10 (1.5)	9 (2.3)	1 (0.4)
Tumor characteristics			
Recurrence type			
Regional recurrence	39 (5.9)	0	39 (14.6)
Distant recurrence	621 (94.1)	392 (100.0)	229 (85.4)
Stage			
Stage I	114 (17.3)	0	114 (42.5)
Stage II	44 (6.7)	0	44 (16.4)
Stage III	110 (16.7)	0	110 (41.0)
Stage IV	392 (59.4)	392 (100.0)	0
Histologic findings			
Adenocarcinoma	344 (52.1)	203 (51.8)	141 (52.6)
Squamous cell	174 (26.4)	92 (23.5)	82 (30.6)
Non–small cell^d^	117 (17.7)	81 (20.7)	36 (13.4)
Large cell	25 (3.8)	16 (4.1)	9 (3.4)
Treatment history			
Received curative-intent therapy for initial LC diagnosis^e^			
Regional recurrence	39 (5.9)	0	39 (14.6)
Yes	174 (26.4)	0	174 (64.9)
No	419 (63.5)	392 (100)	27 (10.0)
Data missing	28 (4.2)	0	28 (10.5)
Smoking-related factors			
Smoking status^f^			
Former smoker	268 (40.6)	157 (40.1)	111 (41.4)
Current smoker	392 (59.4)	235 (59.9)	157 (58.6)
Clinical factors			
Body mass index^g^			
Mean (SD)	26.7 (4.4)	26.4 (4.4)	27.2 (4.5)
Data missing	11 (1.7)	9 (2.3)	2 (0.8)
Disease history			
No	209 (31.7)	127 (32.4)	82 (30.6)
Yes	444 (67.3)	259 (66.1)	185 (69.0)
Data missing	7 (1.1)	6 (1.5)	1 (0.4)

Abbreviations: LC, lung cancer; LDCT, low-dose computed tomography; NLST, National Lung Screening Trial.

^a^ De novo stage IV is defined as patients who initially received diagnoses at stage IV with metastatic lung cancer; recurrent is defined as patients who developed metastatic disease after an initial diagnosis of stage I-III lung cancer.

^b^ Classification of other race includes American Indian or Alaskan Native, Native Hawaiian or Other Pacific Islander, or more than 1 race.

^c^ Classification of not married/not living together includes never married, divorced, separated, or widowed.

^d^ Classification of non–small cell is based on *International Classification of Diseases for Oncology, Third Revision* code 8046 referring to non–small cell carcinoma not further specified to be adenocarcinoma, squamous cell, or 1 of the other more specific non–small cell categories.

^e^ Receipt of curative-intent therapy refers to either surgery or radiation for initial LC among patients with distant recurrence, and nonreceipt refers to any of the following: no therapy received, receiving chemotherapy, or receiving other systemic therapy.

^f^ Smoking status is the status at time of randomization in NLST.

^g^ Body mass index is calculated as weight in kilograms divided by height in meters squared.

### Association Between Metastatic Disease Type and Overall Survival

**Figure 2.**
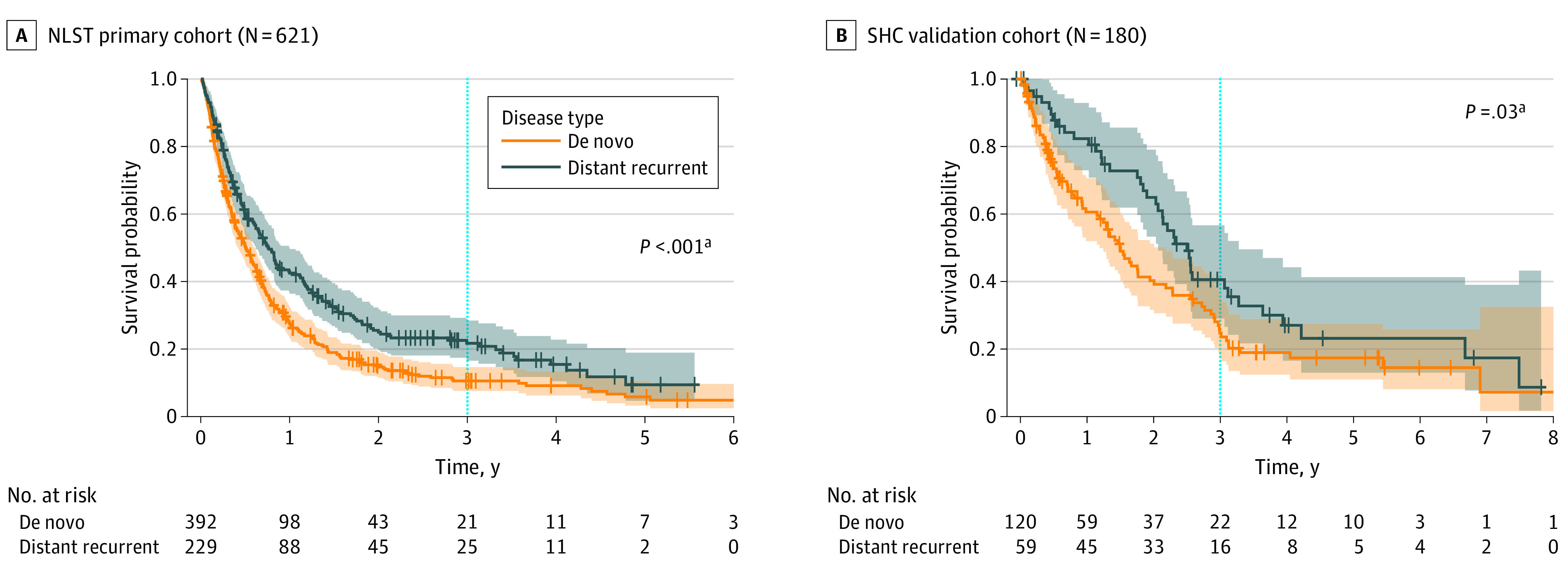
Survival Difference Between Patients With Distant Recurrence and Patients With De Novo Metastatic Disease *P* values are calculated according to hazard ratio and CI adjusted for age, sex, race, and histologic findings of primary lung cancer, with the addition of screening modality and smoking status for the National Lung Screeing Trial (NLST) cohort. SHC indicates Stanford Healthcare. ^a^Sample size in panel A excludes patients with regional recurrence (39 patients) from the entire cohort (660 patients).

The patients with distant recurrence exhibited significantly higher OS than patients with de novo metastatic disease (3-year OS, 21.7% vs 10.5%; adjusted hazard ratio [aHR], 0.72; 95% CI, 0.60-0.87; *P* < .001) (Figure 2A, Table 2; eFigure 3 in Supplement 1), with consistent results when restricting to only those who received curative-intent therapy among patients with distant recurrence (eFigure 4 and eTable 3 in Supplement 1). Sensitivity analysis using PSM were in line with the primary analysis, showing significantly higher survival among patients with distant recurrence (aHR, 0.73; 95% CI, 0.59-0.90; *P* = .004) (eFigure 5 and eTable 4 in Supplement 1). Including patients with regional recurrence, the finding was consistent but with a greater association (3-year OS, 24.1% for patients with recurrence vs 10.5% for patients with de novo metastasis; aHR, 0.68; 95% CI, 0.56-0.82; *P* < .001) compared with the primary analysis, which was expected given the better projected outcomes of patients with regional recurrence (eFigure 6 and eTable 5 in Supplement 1).

**Table 2.** Multivariable Cox Regression to Evaluate Association Between Type of Metastatic Disease and Overall Survival

Covariate	NLST primary cohort^a^		SHC validation cohort	
	Hazard ratio (95% CI)	*P* value	Hazard ratio (95% CI)	*P* value
Type of metastatic disease^b^				
De novo stage IV	1 [Reference]	<.001	1 [Reference]	.03
Distant recurrent	0.72 (0.60-0.87)		0.72 (0.60-0.87)	
Age of metastatic disease	1.01 (1.00-1.03)	.14	1.01 (1.00-1.05)	.04
Sex				
Female	1 [Reference]	.02	1 [Reference]	.28
Male	1.25 (1.03-1.50)		1.25 (0.84-1.86)	
Race				
White	1 [Reference]	.01	1 [Reference]	.01
Black	1.07 (0.70-1.64)		0.53 (0.16-1.74)	
Asian	4.69 (2.29-9.63)		0.98 (0.59-1.64)	
Other^c^	1.19 (0.63-2.24)		2.26 (1.36-3.80)	
Histologic findings				
Adenocarcinoma	1 [Reference]	.19	1 [Reference]	.41
Squamous cell	1.13 (0.91-1.40)		1.18 (0.63-2.20)	
Non–small cell^d^	1.27 (1.00-1.60)	NA	1.31 (0.69-2.41)	
Large cell	1.30 (0.84-2.02)		8.09 (1.04-62.74)	
Screening method				
Chest radiograph	1 [Reference]	.55	NA	NA
LDCT	0.95 (0.79-1.13)		NA	NA
Smoking status^e^				
Former smoker	1 [Reference]	.42	NA	NA
Current smoker	1.08 (0.90-1.30)		NA	NA

Abbreviations: LDCT, low-dose computed tomography; NA, not applicable; NLST, National Lung Screening Trial; SHC, Stanford Healthcare.

^a^ Sample size was 621 participants, which excludes patients with regional recurrence (39 participants) from the entire cohort (660 participants).

^b^ De novo stage IV is defined as patients who initially received diagnoses at stage IV with metastatic lung cancer; distant recurrent is defined as patients who developed distant metastatic disease after an initial diagnosis of stage I to III lung cancer.

^c^ Classification of other race includes American Indian or Alaskan Native, Native Hawaiian or Other Pacific Islander, or more than 1 race.

^d^ Classification of non–small cell for NLST Primary Cohort is based on *International Classification of Diseases for Oncology, Third Revision* code 8046 referring to non–small cell carcinoma not further specified to be adenocarcinoma, squamous cell, or 1 of the other more specific non–small cell categories; for SHC, the validation cohort include all histologic findings other than adenocarcinoma, squamous cell carcinoma, and large cell carcinoma.

^e^ Smoking status is the status at time of randomization in NLST.

### Baseline Characteristics of the SHC Cohort

The SHC validation cohort (180 patients) consisted of 109 male patients (60.6%), 111 White patients (61.7%), 146 patients with adenocarcinoma in histologic findings (81.1%), and a mean (SD) age at time of metastatic disease of 71.4 (7.9) years (eTable 1 in Supplement 1). The second most prevalent racial group was Asian at 18.3% (33 patients). Nearly 70% of patients had de novo stage IV NSCLC (120 patients) and 33.3% had distant recurrence (60 patients), of whom 95.0% had received curative-intent therapy (57 patients). *EGFR* and *KRAS* variations were present in 29 tumors (16.1%) and 58 tumors (32.2%) in this cohort respectively. One-fifth of the SHC cohort (35 patients) had high tumor burden (defined as the top quartile; see Methods), and 30.0% had poor performance status (54 patients; ECOG 2-4) at the time of distant recurrence. Metastasis to the bone made up the highest proportion of patients at 45.6% (82 patients). The median (IQR) follow-up time from diagnosis of metastatic disease to death or censoring was 1.0 (0.4-2.6) years for patients with de novo metastases and 2.1 (1.0-3.1) years for those with distant recurrence (eFigure 7 in Supplement 1).

### Validation and Investigating Underlying Mechanism

The association between survival and metastatic disease type was consistently observed in the SHC cohort as in the NLST cohort. Patients with distant recurrence had significantly higher survival than those with de novo metastasis (aHR, 0.64; 95% CI, 0.43-0.96; *P* = .03) (Figure 2B, Table 2; eFigure 3 in Supplement 1), adjusting for the same covariates used in the NLST cohort analysis, except for the detection mode of primary NSCLC and smoking status. However, these 2 variables were not shown to be independently associated with OS in the multivariable model in the NLST cohort (Table 2). Results were consistent when restricted to receipt of curative-intent therapy (eFigure 4 and eTable 3 in Supplement 1).

When examining additional variables only available in the SHC cohort to compare characteristics by metastatic disease type (Table 3), we found that a substantially higher proportion of the patients with de novo metastasis (41 patients [34.2%] vs 4 patients [6.70%] for distant recurrence) had a high tumor burden. A majority of the patients with de novo metastasis had their metastatic disease detected through symptoms (85.0%) or incidental imaging findings (11.7%), while, as expected, most patients with distant recurrence had their metastatic disease detected through posttreatment surveillance (78.3%). Moreover, patients with de novo metastasis had a notably higher proportion of malignant metastasis to the pleura (33.3% vs 13.3%) and bone (52.5% vs 31.7%) compared with those with distant recurrence.

**Table 3.** Comparison of Clinical Variables by Type of Metastatic Disease in the Validation Stanford Healthcare Cohort

Variable	Type of metastatic disease, No. (%)		*P* value
	De novo stage IV (n = 120)	Distant recurrent (n = 60)	
Presence of prior cancer			
Yes	28 (23.3)	19 (31.7)	.31
No	92 (76.7)	41 (68.3)	
Presence of *EGFR* variant			
Yes	18 (15.0)	11 (18.3)	.72
No	102 (85.0)	49 (81.7)	
Presence of *KRAS* variant			
Yes	44 (36.7)	14 (23.3)	.10
No	76 (63.3)	46 (76.7)	
Metastasis to pleura			
Yes	40 (33.3)	8 (13.3)	.007
No	80 (66.7)	52 (86.7)	
Metastasis to contralateral lung			
Yes	28 (23.3)	13 (21.7)	.95
No	92 (76.7)	47 (78.3)	
Metastasis to adrenal glands			
Yes	15 (12.5)	5 (8.3)	.56
No	105 (87.5)	55 (91.7)	
Metastasis to bone			
Yes	63 (52.5)	19 (31.7)	.01
No	57 (47.5)	41 (68.3)	
Metastasis to liver			
Yes	20 (16.7)	5 (8.3)	.20
No	100 (83.3)	55 (91.7)	
Metastasis to brain			
Yes	42 (35.0)	19 (31.7)	.78
No	78 (65.0)	41 (68.3)	
Received surgery^a^^,^^b^			
Yes	4 (3.3)	0	.37
No	118 (96.7)	60 (100.0)	
Received chemotherapy^a^^,^^b^			
Yes	68 (56.7)	24 (40.0)	.05
No	52 (43.3)	36 (60.0)	
Received radiation^a^^,^^b^			
Yes	18 (15.0)	8 (13.3)	.94
No	102 (85.0)	52 (86.7)	
Received immunotherapy^a^^,^^b^			
Yes	24 (20.0)	13 (21.7)	.95
No	96 (80.0)	47 (78.3)	
Received targeted therapy^a^^,^^b^			
Yes	24 (20.0)	11 (18.3)	.95
No	96 (80.0)	49 (81.7)	
Method of detection for metastatic disease^a^^,^^c^			
CT surveillance^d^	4 (3.3)	47 (78.3)	<.001
Symptom-based	102 (85.0)	12 (20.0)	
Incidental	14 (11.7)	1 (1.7)	
Tumor burden^a^^,^^e^			
High tumor burden	41 (34.2)	4 (6.7)	<.001
Low tumor burden	79 (65.8)	56 (93.3)	
ECOG Performance status^a^^,^^f^			
Good ECOG	72 (60.0)	45 (75.0)	.07
Poor ECOG	48 (40.0)	16 (25.0)	

Abbreviations: CT, computed tomography; ECOG, Eastern Cooperative Oncology Group; *EGFR*, epidermal growth factor receptor.

^a^ Single imputation (eMethods 3 in Supplement 1) was performed for the Stanford Healthcare cohort before analysis due to a missing rate of 2.2% to 23.3% (eTable 1 in Supplement 1).

^b^ Only treatment for the lung at the time of distant metastatic disease is included.

^c^ Method of detection was defined as surveillance if detected on a CT scan for routine follow-up or for assessing treatment response, incidental if detected on a CT scan not indicated for cancer work-up, and symptom-based if detected on interval CT scan primarily according to symptomatic complains.

^d^ CT surveillance is defined as posttreatment surveillance using CT imaging (routine follow-up or assessment for treatment response) for patients who eventually developed distant recurrence and as screening using low-dose CT imaging for patients who eventually developed de novo stage IV disease.

^e^ Tumor burden was defined as the sum of the longest dimension of all cancerous lesions indicated on a radiology report at the time of distant metastasis; high tumor burden was defined as values above the 75th percentile (ie, 141.75 mm), and low otherwise.

^f^ Good ECOG was defined as ECOG 0 to 1; poor ECOG was defined as ECOG 2 to 4.

In our sensitivity analysis further controlling for these additional variables (tumor burden, detection method, and metastatic sites) restricted to those with curative-intent therapy among patients with distant recurrence, we observed a greater association between metastatic disease type and OS (aHR, 0.38; 95% CI, 0.18-0.78; *P* = .009) (eTable 6 in Supplement 1) than the primary analysis.

## Discussion

In this study, we showed that patients with distant recurrent NSCLC have significantly better survival after the diagnosis of metastatic disease compared with those with de novo stage IV NSCLC diagnoses. We used national-level, population-based data with well-curated information on recurrence and long follow-up to evaluate this association and further validated this finding using an independent cohort in the clinical setting. The main result of this study was consistent across these 2 cohorts despite distinct baseline characteristics of the SHC cohort from NLST, with few patients undergoing cancer screening, a higher proportion of stage IV disease, and substantially different racial make-up. The SHC cohort also represents a more modern patient population who may have benefited from recent therapies, which could explain the overall older age at diagnosis for metastatic disease and longer follow-up after metastatic disease. Moreover, our finding was robust across multiple sensitivity analyses, even after adjusting for clinical characteristics associated with metastatic disease type.

The potential reasons for this differential survival outcome in patients with distant recurrence vs de novo stage IV NSCLC could be attributed to differences in metastasis sites, tumor burden, or methods for detecting metastases. We found that a larger proportion of patients with de novo metastasis had more frequent metastasis to the bone or pleura than patients with recurrence. Although both sites represent distinct progression patterns for lung cancer,^18,19^ their differential prevalence is not surprising as prior studies in breast cancer have shown that differing mechanisms for metastatic spread exist by metastatic disease type.^13^ In the context of our main finding—that patients with distant recurrence exhibit better survival than those with de novo disease—pleural and bone metastases have been identified as independent negative prognostic factors,^20,21,22,23^ with prior studies citing the immunosuppressive nature of their tumor microenvironments and, for pleura metastasis, the permeable nature of the pleura cavity that may reduce the efficacy of immunotherapy.^23,24,25,26^ In addition, patients with de novo disease had their metastasis largely detected through symptoms—likely at higher tumor burden—as opposed to posttreatment surveillance, which is more common for patients with distant recurrence and likely resulting in a lower tumor burden at the time of metastasis. Again, high tumor burden is a negative prognostic factor for lung cancer.^27,28,29,30,31,32,33^ Thus, de novo disease is associated with characteristics that may adversely affect OS, which may explain the unique survival profile by metastatic disease type in lung cancer. Nevertheless, it is notable that the association between metastatic disease type and OS remained consistent even after further adjusting for metastatic site, detection method, and tumor burden.

This finding of the present study has important implications. First, most major clinical trials have not distinguished between the types of metastatic disease for lung cancer.^4,5,6,7^ However, results of the present study suggest that the metastatic disease type is a key—and potentially independent—factor associated with outcomes, which, if not considered, could result in a potential imbalance in baseline characteristics. In this case, a stratified randomization design incorporating metastatic disease type could be used, minimizing its potential confounding effect among patients with heterogeneous types of metastasic disease, the composition of which could vary substantially by study sites despite randomization. Alternatively, the trials can adjust for metastatic disease type in multivariable analysis. Second, while higher tumor burden and metastasis to the pleura and bone may be associated with delayed detection among patients with de novo metastasis, they have all been established as independent negative prognostic factors in various treatment settings, including immunotherapy.^19,20,21,22,23,34^ As there is no difference in how patients of different metastatic disease types are currently managed, it may be interesting for future studies to consider using observational data to investigate the effectiveness of first-line treatments by metastatic disease type. Lastly, this factor associated with outcomes should also be accounted for in cost-effectiveness analyses, including microsimulation models where it may have a substantial influence.

To the best of our knowledge, this study presents the first effort evaluating the association of OS by metastatic disease type in 2 distinct patient cohorts and investigating the potential underlying mechanism for survival difference. We chose NLST as a discovery cohort for its large size, national-level, population-based data that includes well-curated information on screening, smoking history, and disease progression separate from subsequent primary cancers. Due to the availability of screening information, we were able to adjust for screening group and detection mode for initial cancer (LDCT vs non-LDCT detected) and found that the main association may potentially be greater among those who underwent LDCT screening, although we did not identify any significant interactions between these factors by metastatic disease type. We then validated our finding using clinical EHR data from a distinct cohort from SHC, a tertiary referral center where patients tend to have more complicated cases as compared with those who receive diagnoses in a screening or trial setting. The recency and granularity of the data enabled the exploration and identification of various clinical characteristics unavailable in NLST data as potential drivers for differential survival by metastatic disease type. The adjustment for these clinical characteristics revealed a consistent association with this study’s main finding. Thus, the use of 2 complementary data sources allowed us to conduct a comprehensive study of survival in metastatic lung cancer, strengthening the robustness of the study finding.

### Limitations

Despite the strengths of the study, a few limitations must be considered. In the NLST cohort, 238 patients were excluded due to unavailable progression data for unknown reasons, which could result in selection bias. Nevertheless, we were able to validate the study finding in an independent cohort. Although the clinical factors identified to possibly explain the survival difference by metastatic disease type were supported by prior literature, the finding may not be generalizable beyond tertiary centers and should be validated in more diverse settings. In the SHC cohort, we could not adjust for primary lung cancer detection method and smoking status due to low LDCT screening rate and lack of updated smoking status. However, these factors were not independently associated with OS in the primary NLST cohort and thus were unlikely to alter our conclusions. Additionally, further research is needed to determine whether individuals with de novo metastasis may have clinical characteristics other than those we investigated that lower the effectiveness of immunotherapy and other therapeutic modalities, but we were unable to do so because of the limited sample size.

## Conclusions

In conclusion, patients with NSCLC with distant recurrence have a significantly higher OS vs patients with de novo metastatic disease from the time of diagnosis of metastatic disease, which was consistent in the clinical setting of SHC. The potential drivers of this differential survival outcome could be attributed to the earlier detection of metastatic disease among patients with distant recurrence through CT surveillance, tumor burden, and metastasis to the pleura and bone. This factor associated with outcomes may have important implications in future clinical trials and cost-effectiveness analyses, and the treatment effectiveness for patients with metastatic NSCLC warrants further evaluation.

## Notes

Supplement 1. **eTable 1. **Characteristics of the SHC Validation Cohort Grouped by Type of Metastatic Disease
eMethods 1.
**eFigure 1.** Overview of Validation Cohort
eMethods 2.

eMethods 3.
**eTable 2.** Characteristics Post-Propensity Score Matching Grouped by Type of Metastatic Disease in the NLST Cohort **eFigure 2.** Distribution of Follow-Up Times **eFigure 3.** Difference in Cumulative Mortality Between Patients With Distant Recurrence and Patients With De Novo Metastatic Disease Among Those With Non–Small Cell Lung Cancer of the NLST Cohort **eFigure 4.** Sensitivity Analysis to Evaluate Overall Survival^1^ Difference Between Patients With De Novo Metastatic Disease Versus Patients With Distant Recurrence Who Received Curative-Intent Therapy During Initial LC Diagnosis **eTable 3.** Sensitivity Analysis Using Multivariable Cox Regression to Evaluate Association Between Type of Metastatic Disease and Overall Survival Comparing Patients With De Novo Stage IV Disease With Subgroup of Distant Recurrent Patients Who Received Curative-Intent Therapy (ie, Surgery or Radiation) in the NLST Primary Cohort and in the SHC Cohort **eFigure 5.** Sensitivity Analysis Using Propensity Score Matching to Evaluate Overall Survival^1^ Difference Between Patients With Distant Recurrence vs De Novo Metastatic Disease in NLST **eTable 4.** Sensitivity Analysis Using Propensity Score Matching to Evaluate the Association Between Type of Metastatic Disease (Distant Recurrent vs De Novo) and Overall Survival Using an Unadjusted Cox Proportional Hazards Mode **eFigure 6.** Sensitivity Analysis to Evaluate Overall Survival^1^ Difference Between Patients With Any Recurrence (Distant or Regional Recurrent) vs Patients With De Novo Metastatic Disease in the Overall NLST Cohort **eTable 5.** Sensitivity Analysis to Evaluate the Association Between any Recurrent (i.e., Regional or Distant Recurrence) vs De Novo Metastatic Disease and Overall Survival in the NLST Cohort **eFigure 7.** Distribution of Follow-Up Times (ie, Time From the Diagnosis of Metastatic Disease to Death or Censoring) in Overall SHC Validation Cohort and by Metastatic Disease Type **eTable 6.** Multivariable Cox Regression to Evaluate Association Between Type of Metastatic Disease (De Novo vs Distant Recurrent) and Overall Survival in the Validation SHC Cohort, Among Patients Who Received Curative Therapy at Initial LC Diagnosis, Additionally Adjusting for Variables That Were Associated With Type of Metastatic Disease in Univariate Analysis

Supplement 2.
Data Sharing Statement
