# Supplementary material for: Overall Survival Among Patients With De Novo Stage IV Metastatic and Distant Metastatic Recurrent Non–Small Cell Lung Cancer
Source: JAMA Netw Open. 2023 Sep 26;6(9):e2335813. doi: 10.1001/jamanetworkopen.2023.35813 (PMC10523163; doi:10.1001/jamanetworkopen.2023.35813)
Supplement: Supplement 1. — eTable 1. Characteristics of the SHC Validation Cohort Grouped by Type of Metastatic Disease eMethods 1. eFigure 1. Overview of Validation Cohort eMethods 2. eMethods 3. eTable 2. Characteristics Post-Propensity Score Matching Grouped by Type of Metastatic Disease in the NLST Cohort eFigure 2. Distribution of Follow-Up Times eFigure 3. Difference in Cumulative Mortality Between Patients With Distant Recurrence and Patients With De Novo Metastatic Disease Among Those With Non–Small Cell Lung Cancer of the NLST Cohort eFigure 4. Sensitivity Analysis to Evaluate Overall Survival1 Difference Between Patients With De Novo Metastatic Disease Versus Patients With Distant Recurrence Who Received Curative-Intent Therapy During Initial LC Diagnosis eTable 3. Sensitivity Analysis Using Multivariable Cox Regression to Evaluate Association Between Type of Metastatic Disease and Overall Survival Comparing Patients With De Novo Stage IV Disease With Subgroup of Distant Recurrent Patients Who Received Curative-Intent Therapy (ie, Surgery or Radiation) in the NLST Primary Cohort and in the SHC Cohort eFigure 5. Sensitivity Analysis Using Propensity Score Matching to Evaluate Overall Survival1 Difference Between Patients With Distant Recurrence vs De Novo Metastatic Disease in NLST eTable 4. Sensitivity Analysis Using Propensity Score Matching to Evaluate the Association Between Type of Metastatic Disease (Distant Recurrent vs De Novo) and Overall Survival Using an Unadjusted Cox Proportional Hazards Mode eFigure 6. Sensitivity Analysis to Evaluate Overall Survival1 Difference Between Patients With Any Recurrence (Distant or Regional Recurrent) vs Patients With De Novo Metastatic Disease in the Overall NLST Cohort eTable 5. Sensitivity Analysis to Evaluate the Association Between any Recurrent (i.e., Regional or Distant Recurrence) vs De Novo Metastatic Disease and Overall Survival in the NLST Cohort eFigure 7. Distribution of Follow-Up Times (ie, Time From the Diagnosis of Metastati [file jamanetwopen-e2335813-s001.pdf]

Supplemental Online Content

Su CC, Wu JT, Choi E, et al. Overall survival among patients with de novo stage IV metastatic and distant metastatic recurrent non–small cell lung cancer. *JAMA Netw Open*. 2023;6(9):e2335813. doi:10.1001/jamanetworkopen.2023.35813

eTable 1. Characteristics of the SHC Validation Cohort Grouped by Type of Metastatic Disease
eMethods 1.
eFigure 1. Overview of Validation Cohort
eMethods 2.
eMethods 3.
eTable 2. Characteristics Post-Propensity Score Matching Grouped by Type of Metastatic Disease in the NLST Cohort
eFigure 2. Distribution of Follow-Up Times
eFigure 3. Difference in Cumulative Mortality Between Patients With Distant Recurrence and Patients With De Novo Metastatic Disease Among Those With Non–Small Cell Lung Cancer of the NLST Cohort
eFigure 4. Sensitivity Analysis to Evaluate Overall Survival[1] Difference Between Patients With De Novo Metastatic Disease Versus Patients With Distant Recurrence Who Received Curative-Intent Therapy During Initial LC Diagnosis
eTable 3. Sensitivity Analysis Using Multivariable Cox Regression to Evaluate Association Between Type of Metastatic Disease and Overall Survival Comparing Patients With De Novo Stage IV Disease to Subgroup of Distant Recurrent Patients Who Received Curative-Intent Therapy (ie, Surgery or Radiation) in the NLST Primary Cohort and in the SHC Cohort.
eFigure 5. Sensitivity Analysis Using Propensity Score Matching to Evaluate Overall Survival[1] Difference Between Patients With Distant Recurrence vs De Novo Metastatic Disease in NLST
eTable 4. Sensitivity Analysis Using Propensity Score Matching to Evaluate the Association Between Type of Metastatic Disease (Distant Recurrent vs De Novo) and Overall Survival Using an Unadjusted Cox Proportional Hazards Mode
eFigure 6. Sensitivity Analysis to Evaluate Overall Survival[1] Difference Between Patients With any Recurrence (Distant or Regional Recurrent) vs Patients With De Novo Metastatic Disease in the Overall NLST Cohort
eTable 5. Sensitivity Analysis to Evaluate the Association Between any Recurrent (i.e., Regional or Distant Recurrence) vs De Novo Metastatic Disease and Overall Survival in the NLST Cohort
eFigure 7. Distribution of Follow-Up Times (ie, Time From the Diagnosis of Metastatic Disease to Death or Censoring) in Overall SHC Validation Cohort and by Metastatic Disease Type
eTable 6. Multivariable Cox Regression to Evaluate Association Between Type of Metastatic Disease (De Novo vs Distant Recurrent) and Overall Survival in the Validation SHC Cohort, Among Patients Who Received Curative Therapy at Initial LC Diagnosis, Additionally Adjusting for Variables That Were Associated With Type of Metastatic Disease in Univariate Analysis

This supplemental material has been provided by the authors to give readers additional information about their work.

© 2023 Su CC et al. *JAMA Network Open.*

eTable 1. Characteristics of the SHC Validation Cohort Grouped by Type of Metastatic Disease

			Type of Metastatic Disease			
	Total		De Novo Stage IV*		Recurrent*	
	N	(%)	N	(%)	N	(%)
**Total No. of Events**	180	(100.0)	120	(66.7)	60	(33.3)
**Follow-up Time (Years)**						
Median (IQR)	1.3	(0.4 – 2.9)	1.0	(0.4 – 2.6)	2.1	(1.0 – 3.1)
**Demographics**						
Age at LC Diagnosis (years)						
Mean (SD)	70.8	(7.9)	71.3	(8.1)	69.7	(7.4)
Age at Diagnosis of Metastatic Disease (years)						
Mean (SD)	71.4	(7.9)	71.3	(8.1)	71.5	(7.5)
Sex						
Female	71	(39.4)	42	(35.0)	29	(48.3)
Male	109	(60.6)	78	(65.0)	31	(51.7)
Race (self-reported)						
Asian	33	(18.3)	22	(18.3)	11	(18.3)
Black	7	(3.9)	4	(3.3)	3	(5.0)
White	111	(61.7)	72	(60.0)	39	(65.0)
Other†	25	(13.9)	19	(15.8)	6	(10.0)
Data missing	4	(2.2)	3	(2.5)	1	(1.7)
**Clinical history**						
Presence of prior cancer before initial lung cancer						
Yes	47	(26.1)	28	(23.3)	19	(31.7)
No	133	(83.9)	92	(76.7)	41	(68.3)
**Tumor Characteristics**						
Initial Stage of Diagnosis						
Stage I	18	(10.0)	0	(0.0)	18	(30.0)
Stage II	17	(9.4)	0	(0.0)	17	(28.3)
Stage III	25	(13.9)	0	(0.0)	25	(41.7)
Stage IV	120	(66.7)	120	(100.0)	0	(0.0)
Histology						
Adenocarcinoma	146	(81.1)	103	(85.8)	43	(71.7)
Squamous cell	19	(10.6)	11	(9.2)	8	(13.3)
Non-small cell‡	14	(7.8)	6	(5.0)	8	(13.3)
Large cell	1	(0.6)	0	(0.0)	1	(1.7)
Presence of EGFR mutation						
Yes	29	(16.1)	18	(15.0)	11	(18.3)
No	151	(83.9)	102	(85.0)	49	(81.7)
Presence of KRAS mutation						
Yes	58	(32.2)	44	(36.7)	14	(23.3)
No	122	(67.8)	76	(63.3)	46	(76.7)
(Continued next page)						
**Sites of Distant Metastasis**						
Metastasis to pleura						

© 2023 Su CC et al. *JAMA Network Open.*

Yes	48	(26.7)	40	(33.3)	8	(13.3)
No	132	(73.3)	80	(66.7)	52	(86.7)
Metastasis to contralateral lung						
Yes	41	(22.8)	28	(23.3)	13	(21.7)
No	139	(77.2)	92	(76.7)	47	(78.3)
Metastasis to adrenal glands						
Yes	20	(11.1)	15	(12.5)	5	(8.3)
No	160	(88.9)	105	(87.5)	55	(91.7)
Metastasis to bone						
Yes	82	(45.6)	63	(52.5)	19	(31.7)
No	98	(54.4)	57	(47.5)	41	(68.3)
Metastasis to liver						
Yes	25	(13.9)	20	(16.7)	5	(8.3)
No	155	(86.1)	100	(83.3)	55	(91.7)
Metastasis to brain						
Yes	61	(33.0)	42	(35.0)	19	(31.7)
No	119	(67.0)	78	(65.0)	41	(68.3)
**Treatments Received for Advanced Disease**						
Received surgery						
Yes	175	(97.2)	115	(95.8)	0	(0.0)
No	1	(0.6)	1	(0.8)	60	(100.0)
Missing	4	(2.2)	4	(3.3)	0	(0.0)
Received chemotherapy						
Yes	90	(50.0)	66	(55.0)	24	(40.0)
No	85	(47.2)	49	(40.8)	36	(60.0)
Missing	5	(2.8)	5	(4.2)	0	(0.0)
Received radiation						
Yes	25	(13.9)	17	(14.2)	8	(13.3)
No	151	(83.9)	99	(82.5)	52	(86.7)
Missing	4	(2.2)	4	(3.3)	0	(0.0)
Received immunotherapy						
Yes	36	(20.0)	23	(19.2)	13	(21.7)
No	140	(77.8)	93	(77.5)	47	(78.3)
Missing	4	(2.2)	4	(3.3)	0	(0.0)
Received targeted therapy						
Yes	32	(17.8)	21	(17.5)	11	(18.3)
No	144	(80.0)	95	(79.2)	49	(81.7)
Missing	4	(2.2)	4	(3.3)	0	(0.0)
Received curative-intent therapy (surgery or radiation)						
Yes	57	(31.7)	0	(0.0)	57	(95.5)
No	119	(66.1)	116	(96.7)	3	(5.0)
Missing	4	(2.2)	4	(33.3)	0	(0.0)
(Continued next page)						
**Method of detection for metastatic disease§**						
CT surveillance	50	(27.8)	4	(3.3)	46	(76.7)

© 2023 Su CC et al. *JAMA Network Open.*

Incidental	14	(7.8)	13	(10.8)	1	(1.7)
Symptom-based	109	(60.6)	98	(81.7)	11	(18.3)
Missing	7	(3.9)	5	(4.2)	2	(3.3)
**Disease Severity**						
**Tumor burden††**						
High tumor burden	35	(19.4)	31	(25.8)	4	(6.7)
Low tumor burden	103	(57.2)	60	(50.0)	43	(72.7)
Missing	42	(23.3)	29	(24.2)	13	(21.7)
**ECOG Performance status (grouped)**						
Good ECOG	109	(60.6)	68	(56.7)	41	(68.3)
Poor ECOG	54	(30.0)	40	(33.3)	14	(23.3)
Missing	17	(9.4)	12	(10.0)	5	(8.3)
**ECOG Performance status (ungrouped)**						
0	31	(17.2)	20	(16.7)	11	(18.3)
1	78	(43.3)	48	(40.0)	30	(50.0)
2	36	(20.0)	24	(20.0)	12	(20.0)
3	16	(8.9)	14	(11.7)	2	(3.3)
4	2	(1.1)	2	(1.7)	0	(0.0)
Missing	17	(9.4)	12	(10.0)	5	(8.3)

Abbreviations. SHC, Stanford Healthcare; IQR, Interquartile Range; SD, Standard Deviation; ECOG, Eastern Cooperative Oncology Group.
Note: Highlighted variables were variables used as covariates in the primary analysis, and thus again included in the validation analysis.
* De novo Stage IV is defined as patients who were initially diagnosed at Stage IV with metastatic lung cancer; Recurrent is defined as patients who developed metastatic disease after an initial diagnosis of Stage I-III lung cancer.
† Classification of 'other' race include, Native Hawaiian or Other Pacific Islander, and all other race groups.
‡ Classification of 'Non-small cell' include all histology types other than adenocarcinoma, squamous cell carcinoma, and large cell carcinoma.
†† Tumor burden was defined as the sum of the longest dimension of all cancerous lesions indicated on a radiology report at the time of distant metastasis; High tumor burden was defined as values above the 75th percentile (i.e., 141.75mm), and low otherwise.
** Good ECOG was defined as ECOG 0-1, Poor ECOG was defined as ECOG 2-4.

© 2023 Su CC et al. *JAMA Network Open.*

eMethods 1.

Data Curation for Stanford Healthcare Cohort

Data was manually curated by a senior graduate student in collaboration with a medical oncologist. 200 patients were randomly selected from the initial cohort of 547 patients with metastatic disease and molecular profiling at SHC. Patients were defined to have "De novo Stage IV" metastatic disease if they were diagnosed at Stage IV and to have "Distant Recurrent" metastatic disease if they had recurrence to pleura, contralateral lung, skin, adrenal, bone, liver, brain and/or any other distant organs outside the chest at the time of diagnosis for distant metastatic disease. All data collected was manually collected in RedCap. The Stanford University IRB approved this study, and a waiver of informed consent was received as the study presented a minimal risk.

ECOG performance status was obtained from the recorded value in an oncology progress note at the time of distant metastatic disease. This variable is recoded into a binary variable of good versus poor performance status groups, where ECOG 0-1 was considered good performance status while ECOG 2-4 was considered poor performance status.

Method of detection for initial primary lung cancer was curated based on clinical history in progress notes. It is defined as 'surveillance' if detected on a CT scan for routine follow-up or for assessing treatment response for those who eventually developed distant recurrence or if detected on a low-dose CT screening for those who eventually developed de novo disease, 'incidental' if detected on a CT scan not indicated for cancer work-up, and 'symptom-based' if detected on interval CT scan/LDCT screening primarily based on symptomatic complains.

Tumor burden was curated from radiology report nearest to the date of diagnosis for metastatic disease selected in the following priority: 1) CT Chest Abdomen Pelvis, 2) CT Abdomen Liver with IV Contrast Triphasic Chest Pelvis, 3) CT Chest + CT Abdomen Pelvis, 4) PET-CT, 5) CT Chest or CT Thorax, if more than one is present within 30 days before or after the date of diagnosis. Tumor burden is defined as the sum of the longest dimension of all cancerous lesions indicated on a radiology report at the time of distant metastasis.[17] This measure was categorized into low versus high groups where it is considered high if above the 75th percentile (i.e., 141.75 mm) of the entire cohort and low if otherwise.

All other variables curated (e.g., driver mutations present, treatments for metastatic disease, and site of metastatic disease at the time of diagnosis of distant metastatic disease) were curated from oncology progress notes and their categories are listed in **Supplementary Table S1**.

eFigure 1. Overview of Validation Cohort
At SHC, 547 patients with metastatic NSCLC who have molecular profiling were identified. Of these patients, 200 patients were randomly selected to undergo manual chart review. After data curation, the validation cohort consisted of 180 patients with confirmed distant metastasis.

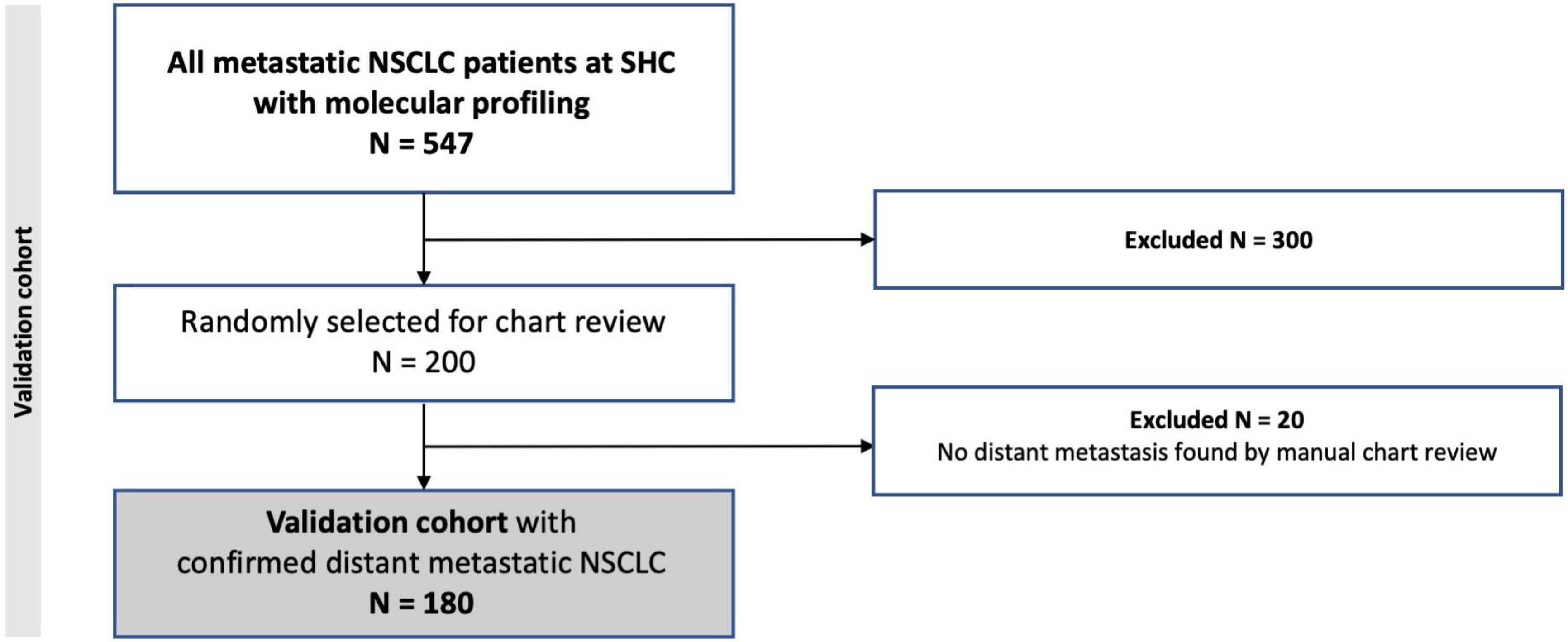

Figure 1 footnote:
Abbreviations. SHC, Stanford Healthcare; NSCLC, Non-Small Cell Lung Cancer.

© 2023 Su CC et al. *JAMA Network Open.*

eMethods 2.

1. Propensity Score Matching

Calculating Propensity Score

In a clinical study, propensity score is an estimated probability of a patient being in the exposed group given the covariates that represent the characteristics of the patient, as opposed to the unexposed group. The goal of the propensity score is to account for potential confounders, and it is most often applied in observational studies when randomization did not or cannot take place. In these studies, the exposed and unexposed groups may not be comparable due to inherent differences resulting in being in one group or the other. For example, a patient who received surgery (thus in the exposed group), may have undergone surgery because of better medical condition and prognosis. The goal of calculating propensity scores is to balance measured potential confounders and make the two groups comparable.

The propensity score is calculated using logistic regression. This is the probability of being in the exposed group given the covariates being balanced (Eqn. 1). The logit model is built with the exposure as the binary outcome and the potential confounders as covariates (Eqn. 2). The model yields a probability of being in the exposed group for each subject in the study cohort. With this method, a large number of covariates can be reduced to a single probability.

$P(Distant\ recurrence \mid Covariates)$ ---- **Eqn. 1**

$Logit\ (Distant\ recurrence) = \alpha + \beta_{covariate\ 1} + \beta_{covariate\ 2} + \cdots$ ---- **Eqn. 2**

In this study, the “exposed” group consists of patients with distant recurrence and the “unexposed” group consists of patients with de novo Stage IV metastatic Non-Small Cell Lung Cancer (NSCLC). In our propensity score model, we included both variables that are already adjusted for in the competing risk regression models (i.e., age of LC diagnosis, sex, race, histology of NSCLC, screening method and smoking status) and additional variables that may be potential confounders and were previously used in propensity score matching by other studies for overall survival after diagnosis of metastatic disease. These additional variables include comorbid disease history and marital status. Comorbid disease history is defined by presence of one or more of the following diseases: asthma, asbestosis, bronchiectasis, childhood asthma, chronic bronchitis, COPD, diabetes, emphysema, fibrosis of the lung, heart disease or heart attack, hypertension, pneumonia, sarcoidosis, silicosis, stroke, tuberculosis. Marital status comprises of either married/living together or not married/not living together. Those who are married were

© 2023 Su CC et al. *JAMA Network Open.*

considered as the former category, and those who are divorced, separated, widowed or never married were considered the latter category. To estimate the propensity score, we fitted logistic regression using type of metastatic disease as an outcome variable and the above listed variables as independent variables.

Matching

Subjects with similar propensity scores are theoretically interchangeable. If two subjects have similar propensity scores but are in different exposure groups, then any difference in outcome could be attributed to the exposure. In our study, we have matched subjects in the distant recurrence group and subjects in the de novo metastatic group in a 1:1 ratio based on their propensity scores. After propensity score matching, we ensured that all the covariates are balanced using Chi-square test or Fisher's exact test (sparse variables) for categorical variables, and the t-test (parametric) or Man Whitney U test (non-parametric) for continuous variables.

eMethods 3.

2. Single imputation

Single imputation was performed by chain equations for missing data as the rate of missingness range from 2.2% to 23.3%, using the full set of variables curated through manual chart review. In addition to the variables listed in **Supplementary Table S2**, other variables included for single imputation are: presence of smoking history, method of detection for initial primary lung cancer (LDCT screening, symptom-based or incidental), index tumor size at initial diagnosis, presence of RET fusion mutation, presence of NTRK3 fusion mutation, presence of ROS1 fusion mutation, presence of ERBB2 mutation, presence of ALK mutation, presence of MET mutation, presence of BRAF mutation, treatment received for initial lung cancer (for patients with distant recurrence), distant metastasis to other sites not listed in Table S2, treatment received for advanced disease not listed in Table S2.

© 2023 Su CC et al. *JAMA Network Open.*

eTable 2. Characteristics Post-Propensity Score Matching Grouped by Type of Metastatic Disease* in the NLST Cohort

	Type of Metastatic Disease						
	Total		De Novo Stage IV*		Distant Recurrent*		
	N	(%)	N	(%)	N	(%)	P-value
**Total No. of Events**	434	(100.0)	217	(50.0)	217	(50.0)	
**Screening Information**							
Screening Method in NLST							0.63
LDCT	234	(53.9)	120	(55.3)	114	(52.5)	
Chest X-Ray	200	(46.1)	97	(44.7)	103	(47.5)	
**Demographics**							
Age at Diagnosis of Metastatic Disease (years)							0.89
Mean (SD)	66.8	(5.7)	66.9	(5.6)	66.8	(5.7)	
Sex							0.77
Female	162	(37.3)	79	(36.4)	83	(38.2)	
Male	272	(62.7)	138	(63.6)	134	(61.8)	
Race							0.99
White	399	(91.9)	200	(92.2)	199	(91.7)	
Black	20	(4.6)	10	(4.6)	10	(4.6)	
Asian	6	(1.4)	3	(1.4)	3	(1.4)	
Other†	9	(2.1)	4	(1.8)	5	(2.3)	
Marital Status							0.53
Married/Living together	299	(68.9)	153	(70.5)	147	(67.7)	
Not married/Not living together‡	135	(31.1)	64	(29.5)	70	(32.3)	
**Tumor Characteristics**							
Histology							0.50
Adenocarcinoma	232	(53.5)	110	(50.7)	122	(56.2)	
Squamous cell	120	(27.6)	61	(28.1)	59	(27.2)	
Non-small cell§	63	(14.5)	34	(15.7)	29	(13.4)	
Large cell	19	(4.4)	12	(5.5)	7	(3.2)	
**Smoking-related Factors**							
Smoking status††							1.00
Former Smoker	182	(41.9)	91	(41.9)	91	(41.9)	
Current Smoker	252	(58.1)	126	(58.1)	126	(58.1)	
**Clinical Factors**							
Disease History							0.61
No	146	(33.6)	76	(35.0)	70	(32.3)	
Yes	288	(66.4)	141	(65.0)	147	(67.7)	

Abbreviations. LDCT, Low Dose Computed Tomography; SD, Standard Deviation.

* De novo Stage IV is defined as patients who were initially diagnosed at Stage IV with metastatic lung cancer; Recurrent is defined as patients who developed metastatic disease after an initial diagnosis of Stage I-III lung cancer.

† Classification of 'other' race include American Indian or Alaskan Native, Native Hawaiian or Other Pacific Islander, More than one race.

‡ Classification of 'Not married/Not living together' include never married, divorced, separated, widowed.

§ Classification of 'Non-small cell' based on ICD-O-3 code 8046 referring to non-small cell carcinoma not further specified to be adenocarcinoma, squamous cell, or one of the other more specific non-small cell categories.

†† Smoking status is the status at time of randomization in NLST.

© 2023 Su CC et al. *JAMA Network Open.*

eFigure 2. Distribution of Follow-Up Times (i.e., time from the diagnosis of metastatic disease to death or censoring) in overall cohort in NLST and by metastatic disease type. The overall cohort (N=660) has a median follow-up time of 0.5 years (IQR: 0.2 – 1.3), in **Panel A**, and the different metastatic disease types each has a median follow-up time of 0.5 years (IQR: 0.2 – 1.0) for patients with de novo metastatic disease, 0.7 years (IQR: 0.3 – 1.6) for patients with distant recurrence and 0.7 years (IQR: 0.3 – 2.1) for patients with regional recurrence, in **Panel B**.

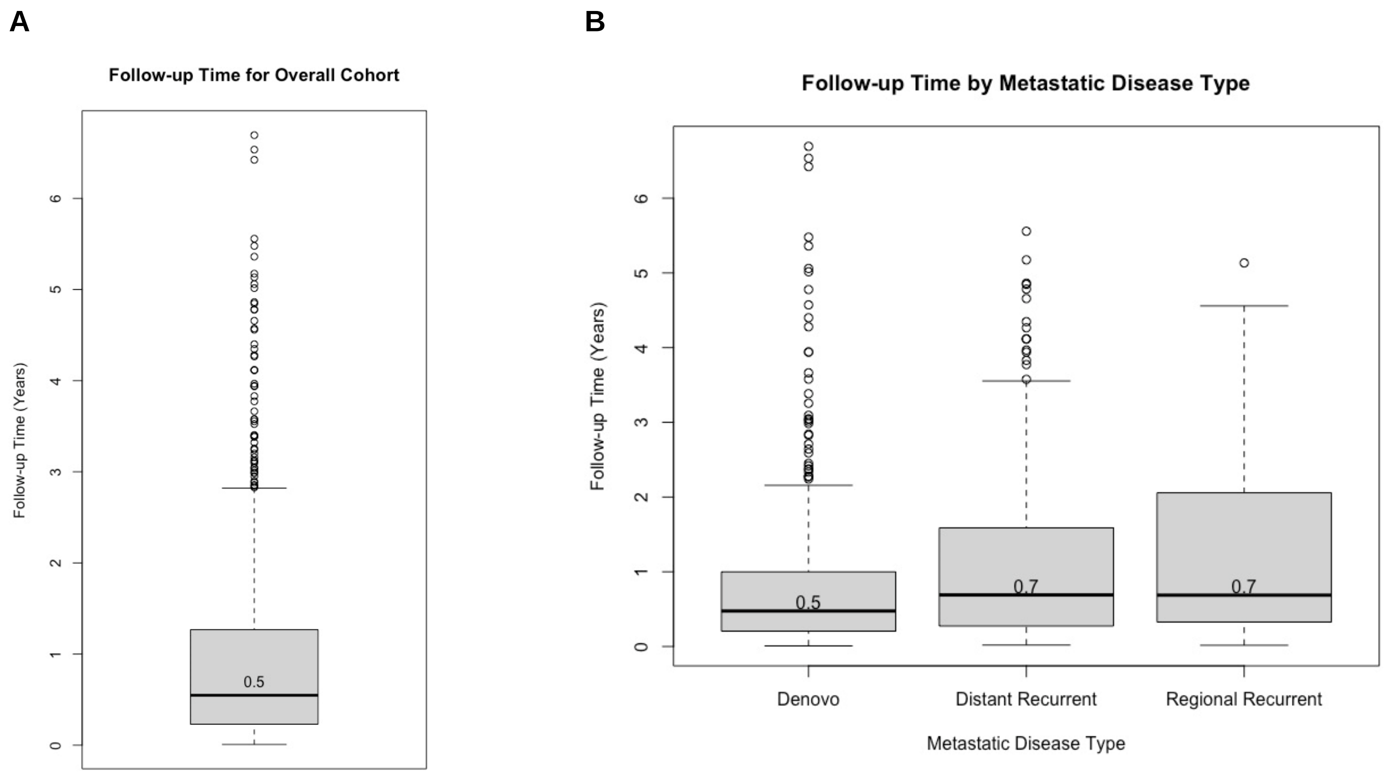

© 2023 Su CC et al. *JAMA Network Open.*

eFigure 3. Difference in **Cumulative Mortality** Between Patients With Distant Recurrence and Patients With De Novo Metastatic Disease Among Those With Non–Small Cell Lung Cancer of the NLST Cohort (N = 621)[a], in A, and the SHC cohort (N = 180), in B.

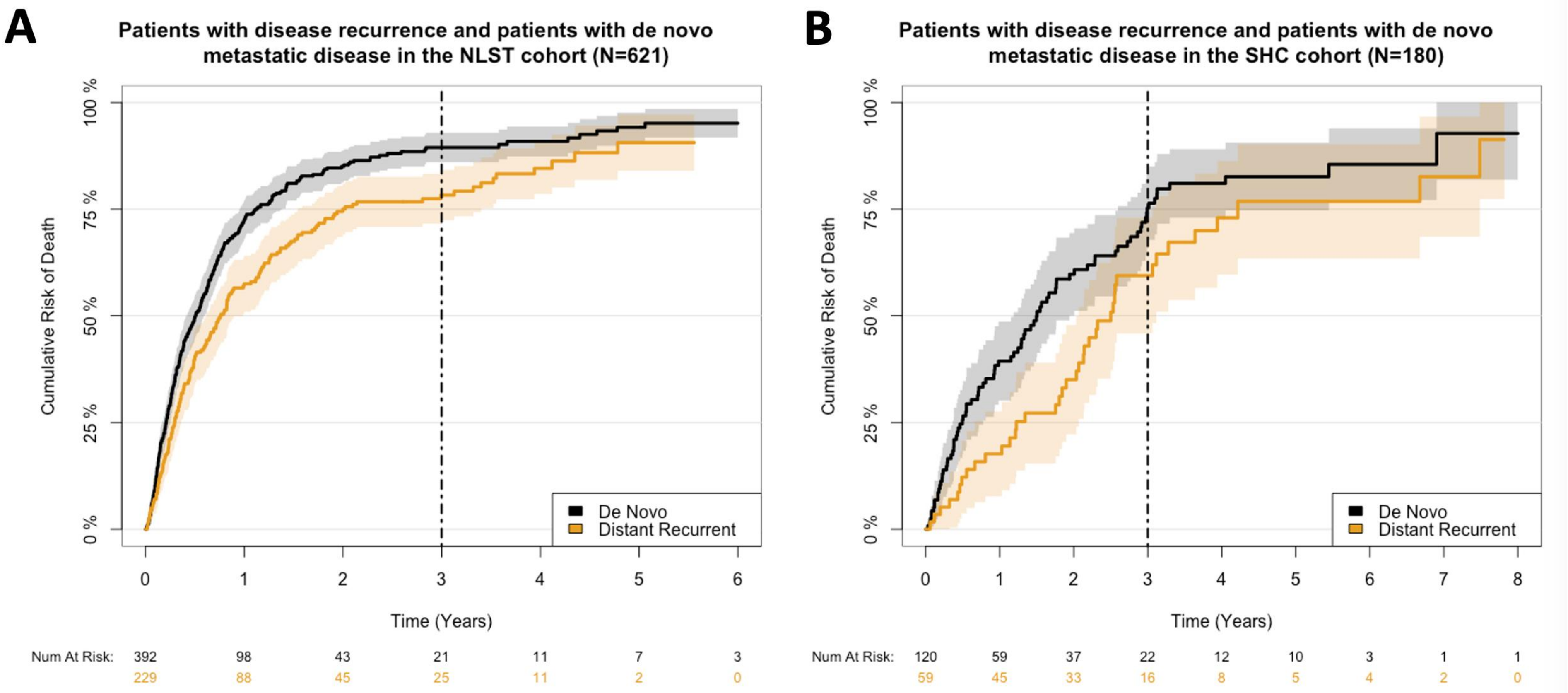

[a] Sample size excludes patients with regional recurrence (N=39) from the entire cohort (N=660).

© 2023 Su CC et al. *JAMA Network Open.*

eFigure 4. Sensitivity Analysis to Evaluate Overall Survival[1] Difference Between Patients Who De Novo Metastatic Disease Versus Patients With Distant Recurrence Who **Received Curative-Intent Therapy** During Initial LC Diagnosis

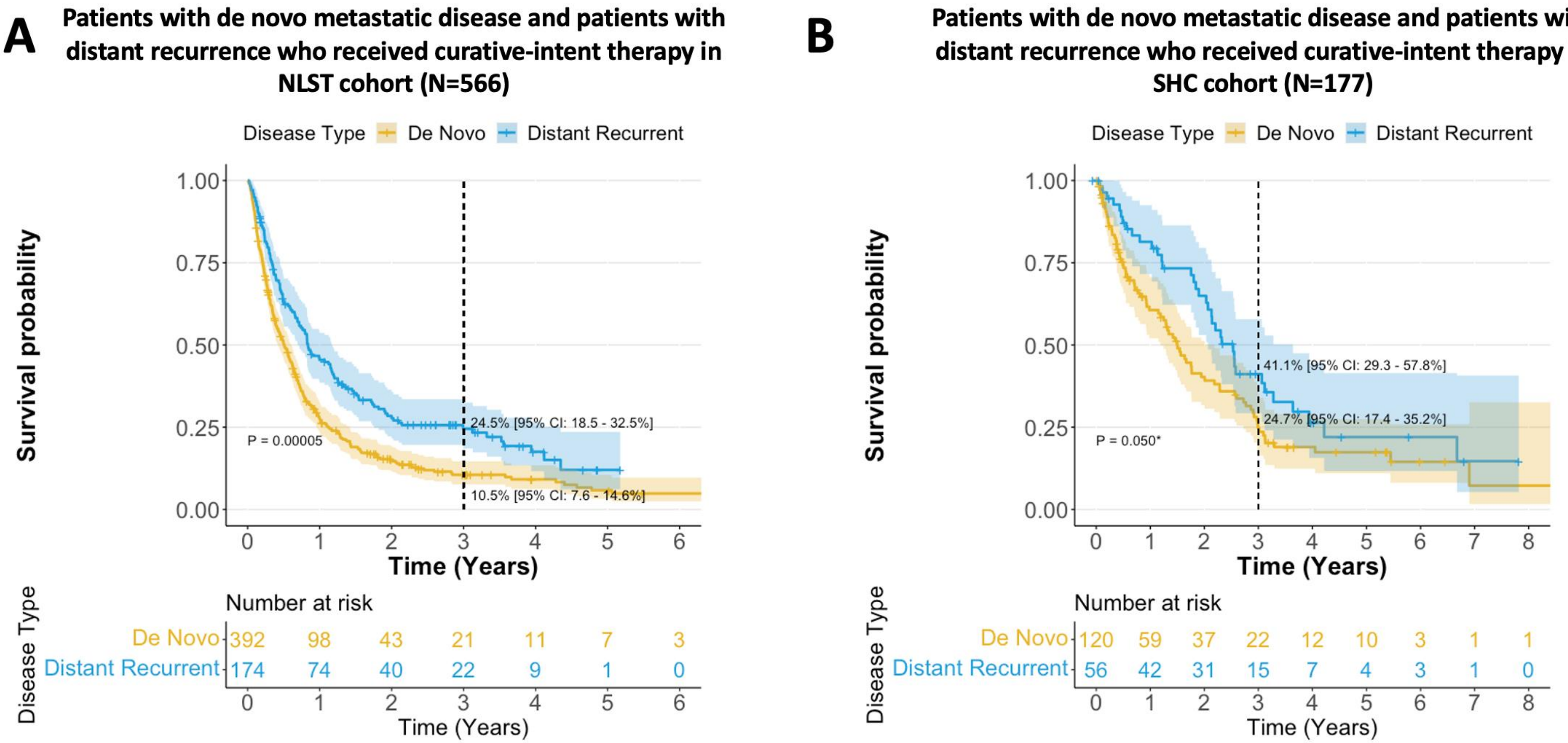

[1] Overall survival was calculated from the time of first distant metastatic disease.
* P-value was calculated based on hazard ratio and confidence interval adjusted for age, sex, race, histology of primary lung cancer, smoking status and screening modality.

© 2023 Su CC et al. *JAMA Network Open.*

eTable 3. Sensitivity Analysis Using Multivariable Cox Regression to Evaluate Association Between Type of Metastatic Disease and Overall Survival Comparing Patients With De Novo Stage IV Disease to Subgroup of distant Recurrent Patients Who Received Curative-Intent Therapy (ie, Surgery or Radiation) in the NLST Primary Cohort (N=566) and in the SHC Cohort (N=177).

	NLST Primary Cohort[a]			SHC Validation Cohort[a]		
Covariate	Hazard Ratio	(95% CI)	P-value	Hazard Ratio	(95% CI)	P-value
**Type of metastatic disease[b]**			< 0.0001			0.050
De novo Stage IV	Ref.	-		Ref.	-	
Distant Recurrent	0.65	(0.52 – 0.80)		0.66	(0.44 – 1.00)	
**Age of Metastatic Disease**	1.01	(0.99 – 1.03)	0.297	1.03	(1.00 – 1.05)	0.035
**Sex**			0.012			0.322
Female	Ref.	-		Ref.	-	
Male	1.29	(1.05 – 1.57)		1.23	(0.82 – 1.84)	
**Race**			0.014			0.037
White	Ref.	-		Ref.	-	
Black	0.93	(0.57 – 1.50)		0.53	(0.16 – 1.72)	
Asian	5.08	(2.47 – 10.47)		0.98	(0.59 – 1.63)	
Other[c]	1.14	(0.58 – 2.22)		2.20	(1.30 – 3.70)	
**Histology**			0.505			0.417
Adenocarcinoma	Ref.	-		Ref.	-	
Squamous cell	1.04	(0.89 – 1.39)		1.15	(0.62 – 2.15)	
Non-small cell[d]	1.16	(0.90 – 1.49)		1.28	(0.66 – 2.46)	
Large cell	1.29	(0.82 – 2.02)		7.75	(1.00 – 60.06)	
**Screening Method**			0.616			
Chest X-Ray	Ref.	-				
LDCT	0.95	(0.79 – 1.15)				
**Smoking Status[e]**			0.405			
Former Smoker	Ref.	-				
Current Smoker	1.09	(0.90 – 1.32)				

Abbreviations. NLST, National Lung Screening Trial; LC, Lung cancer; NOS, Not otherwise specified; LDCT, Low Dose Computed Tomography; SHC: Stanford Healthcare.
[a] NLST cohort sample size = 566, excludes patients with regional recurrence (N=39), patients with distant recurrence who did not receive definitive therapy (N=27), and patients who had missing treatment information (N=28) from the entire cohort (N=660); SHC cohort sample size = 177, excludes patients who received only systemic chemotherapy for initial lung cancer diagnosis (N=3).
[b] De novo Stage IV is defined as patients who were initially diagnosed at Stage IV with metastatic lung cancer; Distant recurrent is defined as patients who developed distant metastatic disease after an initial diagnosis of Stage I-III lung cancer.
[c] Classification of 'other' race include American Indian or Alaskan Native, Native Hawaiian or Other Pacific Islander, More than one race.
[d] Classification of 'Non-small cell' in the NLST Primary Cohort is based on ICD-O-3 code 8046 referring to non-small cell carcinoma not further specified to be adenocarcinoma, squamous cell, or one of the other more specific non-small cell categories; for the SHC Validation cohort includes all histology types other than adenocarcinoma, squamous cell carcinoma, and large cell carcinoma.
[e] Smoking status is the status at time of randomization in NLST.

© 2023 Su CC et al. *JAMA Network Open.*

eFigure 5. Sensitivity Analysis **Using Propensity Score Matching** to Evaluate Overall Survival[1] Difference Between Patients With Distant Recurrence vs De Novo Metastatic Disease in NLST (N=434).

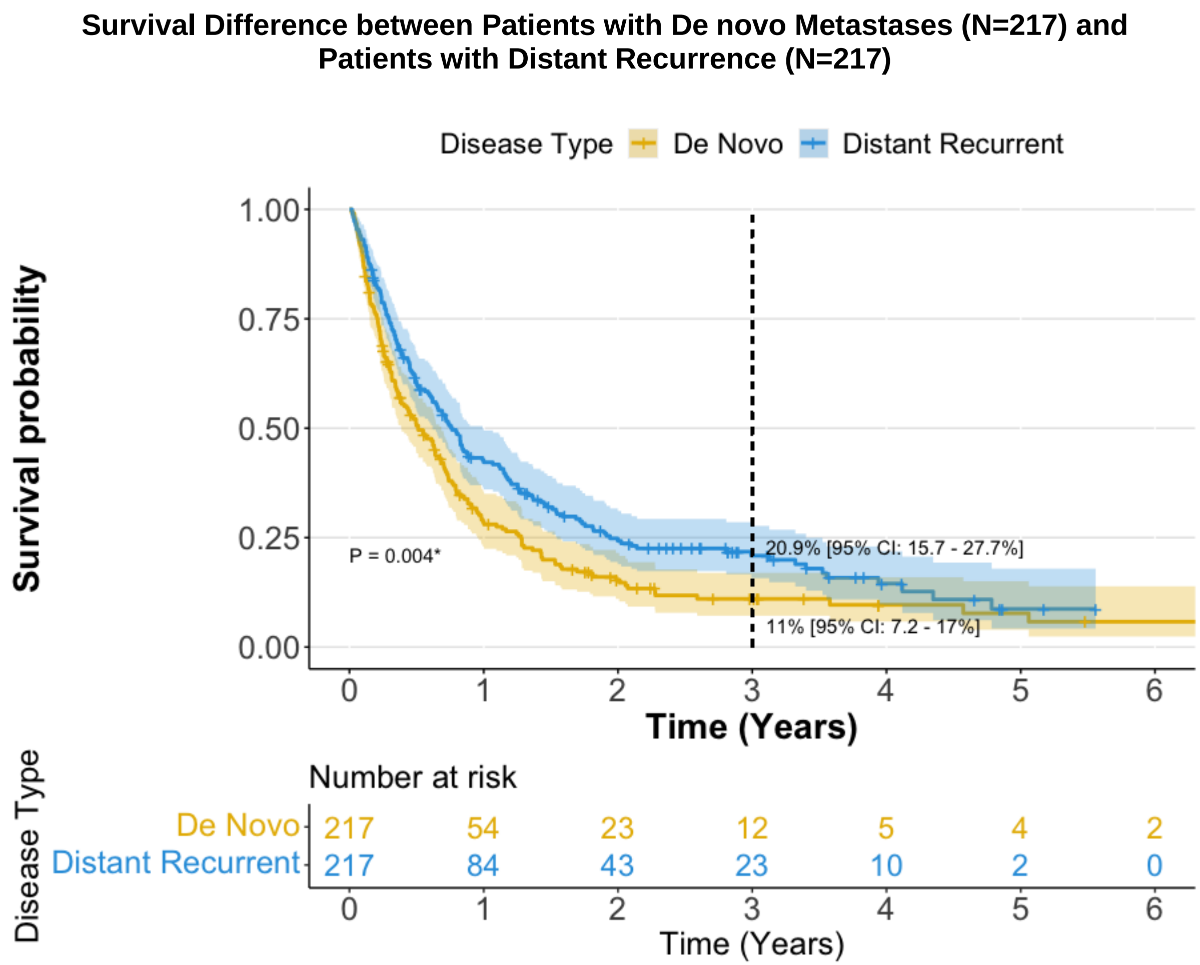

[1] Overall survival was calculated from the time of first distant metastatic disease.
* P-value was calculated based on hazard ratio and confidence interval adjusted for age, sex, race, histology of primary lung cancer, smoking status and screening modality.

© 2023 Su CC et al. *JAMA Network Open.*

eTable 4. Sensitivity Analysis Using Propensity Score Matching (n=434) to Evaluate the Association Between Type of Metastatic Disease (Distant Recurrent vs De Novo) and Overall Survival Using an Unadjusted Cox Proportional Hazards Model. The propensity score based matched patient characteristics are shown in **Table S2,** and the relevant methods are described in **Supplementary Methods 2**.

Covariate	Hazard Ratio	(95% CI)	P-value
**Type of metastatic disease†**			
De novo Stage IV	Ref.		-
Distant Recurrence	0.73	(0.59 – 0.90)	0.004

† De novo Stage IV is defined as patients who were initially diagnosed at Stage IV with metastatic lung cancer; Recurrent is defined as patients who developed metastatic disease after an initial diagnosis of Stage I-III lung cancer.

© 2023 Su CC et al. *JAMA Network Open.*

eFigure 6. Sensitivity Analysis to Evaluate Overall Survival[1] Difference Between Patients With **any Recurrence (Distant or Regional Recurrent)** vs Patients With De Novo Metastatic Disease in the Overall NLST Cohort (N=660**).

Survival Difference between Patients with De novo Metastases (N=392) and Patients with Any Recurrence (N=268)

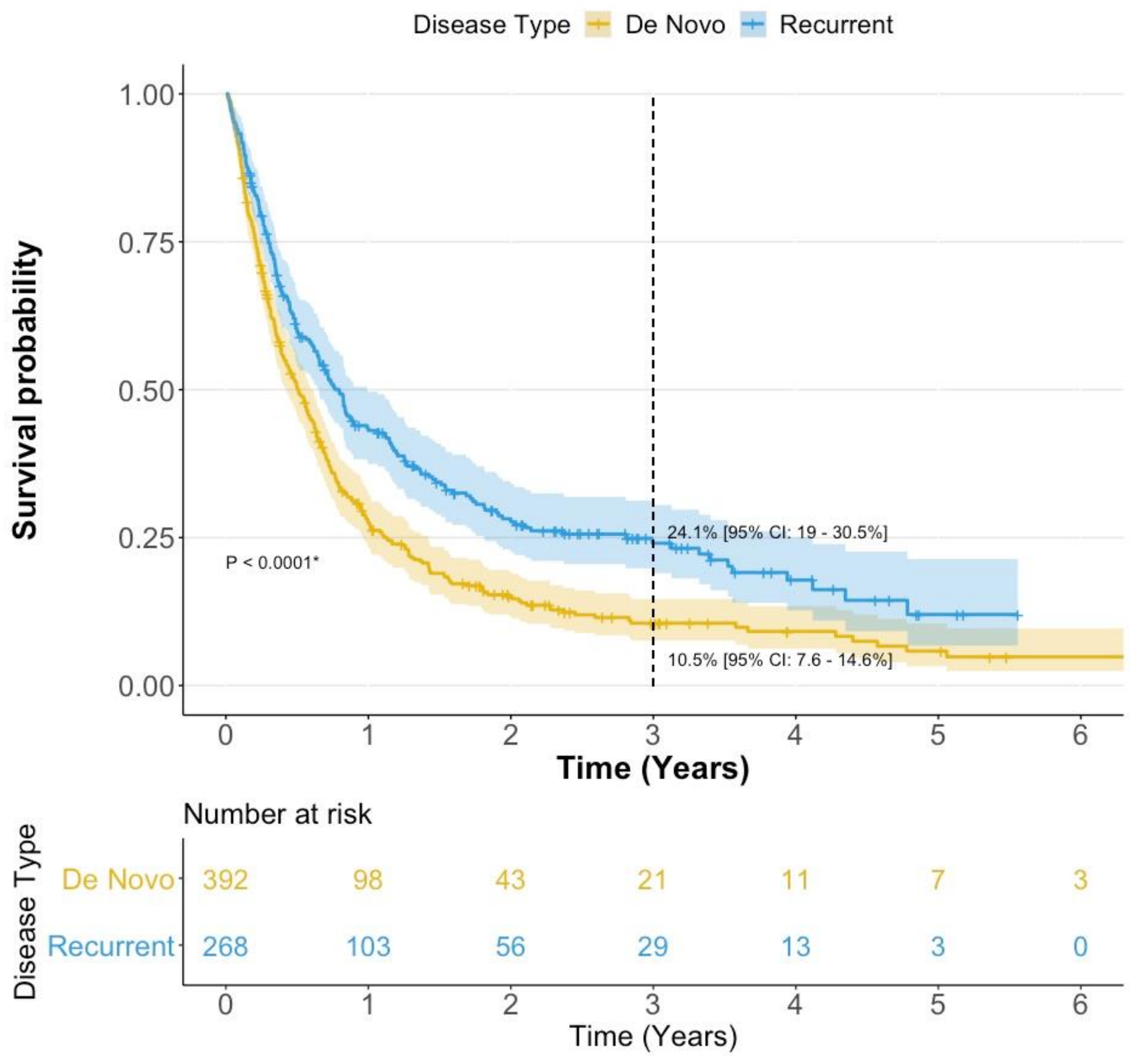

[1] Overall survival was calculated from the time of first metastatic disease (distant or regional).

* P-value was calculated based on hazard ratio and confidence interval adjusted for age, sex, race, histology of primary lung cancer, smoking status and screening modality.

** Sample size includes all patients in the study cohort (regional recurrent, distant recurrent and de novo Stage IV).

© 2023 Su CC et al. *JAMA Network Open.*

eTable 5. Sensitivity Analysis to Evaluate the Association Between any Recurrent (i.e., Regional or Distant Recurrence) vs De Novo Metastatic Disease (N = 660) and Overall Survival in the NLST Cohort.

**Covariate**	**Hazard Ratio**	**(95% CI)**	**P-value**
**Type of metastatic disease†**			<0.0001
De novo Stage IV	Ref.		
Distant Recurrence	0.68	(0.57 – 0.82)	
**Age of Metastatic Disease**	1.02	(0.99 – 1.03)	0.081
**Sex**			0.009
Female	Ref.		
Male	1.27	(1.06 – 1.53)	
**Race**			0.010
White	Ref.		
Black	1.19	(0.79 – 1.80)	
Asian	4.58	(2.24 – 9.39)	
Other‡	1.16	(0.62 – 2.18)	
**Histology**			0.193
Adenocarcinoma	Ref.		
Squamous	1.18	(0.96 – 1.45)	
Non-small cell§	1.24	(0.98 – 1.57)	
Large cell	1.24	(0.80 – 1.97)	
**Smoking Status††**			0.333
Former Smoker	Ref.		
Current Smoker	1.09	(0.91 – 1.31)	
**Screening Method**			0.863
Chest X-Ray	Ref.		
LDCT	0.98	(0.82 – 1.18)	

Abbreviations. LC, Lung cancer.

† De novo Stage IV is defined as patients who were initially diagnosed at Stage IV with metastatic lung cancer; Recurrent is defined as patients who developed metastatic disease (regional or distant) after an initial diagnosis of Stage I-III lung cancer.

‡ Classification of ‘other’ race include American Indian or Alaskan Native, Native Hawaiian or Other Pacific Islander, More than one race.

§ Classification of ‘Non-small cell’ based on ICD-O-3 code 8046 referring to non-small cell carcinoma not further specified to be adenocarcinoma, squamous cell, or one of the other more specific non-small cell categories.

†† Smoking status is the status at time of randomization in NLST.

© 2023 Su CC et al. *JAMA Network Open.*

eFigure 7. Distribution of Follow-Up Times (ie, Time From the Diagnosis of Metastatic Disease to Death or Censoring) in Overall SHC Validation Cohort and by Metastatic Disease Type. The overall validation cohort (N=180) has a median follow-up time of 1.3 years (IQR: 0.4 – 2.9), in **Panel A**, and the different metastatic disease types each has a median follow-up time of 1.0 years (IQR: 0.4 – 2.6) for patients with de novo metastatic disease, and 2.1 years (IQR: 1.0 – 3.1) for patients with distant recurrence, in **Panel B**.

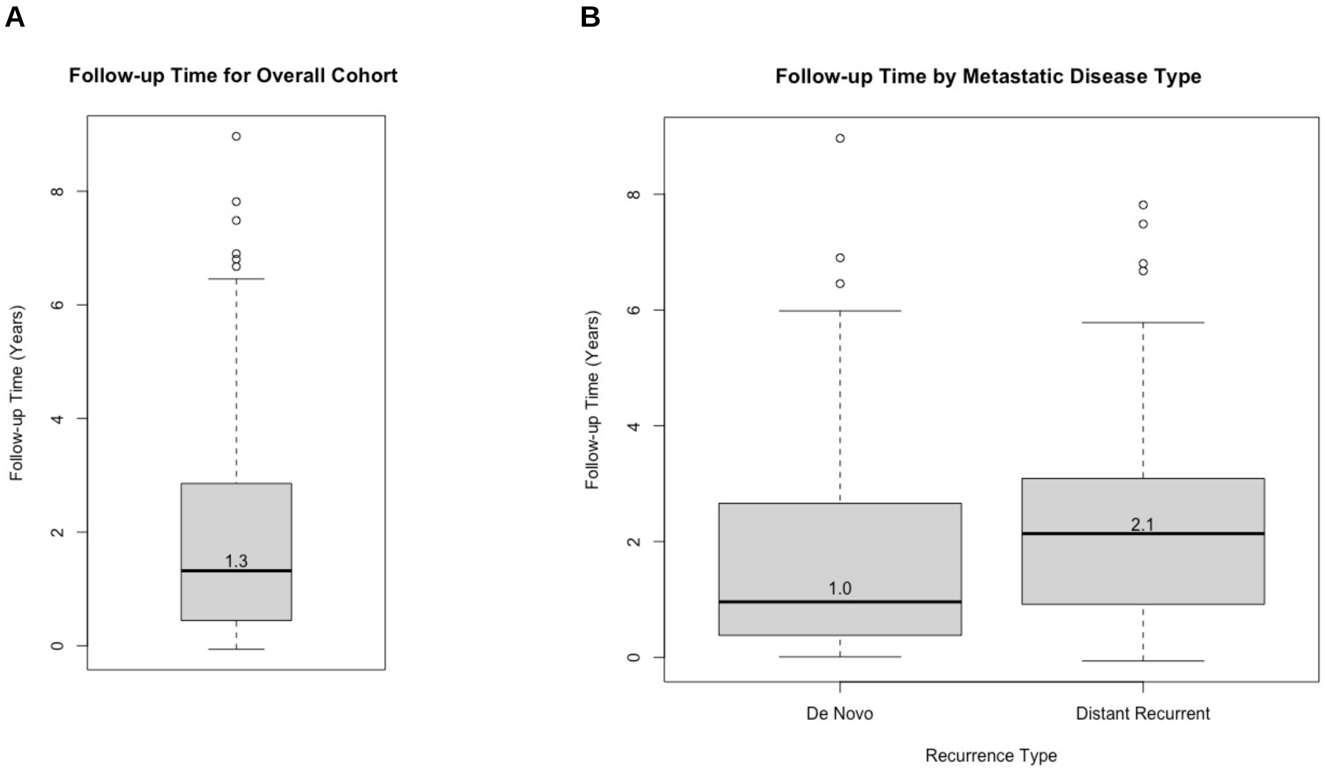

© 2023 Su CC et al. *JAMA Network Open.*

eTable 6. Multivariable Cox Regression to Evaluate Association Between Type of Metastatic Disease (De Novo vs Distant Recurrent) and Overall Survival in the Validation SHC Cohort (N=177), **Amongst Patients Who Received Curative Therapy at Initial LC Diagnosis**, **Additionally Adjusting for Variables That Were Associated With Type of Metastatic Disease in Univariate Analysis** (**Table 3**).

Covariate	Hazard Ratio	(95% CI)	P-value
**Type of metastatic disease†**			0.009
De novo Stage IV	Ref.	-	
Distant Recurrent	0.38	(0.18 – 0.78)	
**Age of Metastatic Disease**	1.03	(1.00 – 1.06)	0.030
**Sex**			0.325
Female	Ref.	-	
Male	1.22	(0.82 – 1.81)	
**Race**			0.004
White	Ref.	-	
Black	0.81	(0.14 – 1.54)	
Asian	0.46	(0.48 – 1.37)	
Other‡	2.39	(1.40 – 4.08)	
**Histology**			0.238
Adenocarcinoma	Ref.	-	
Squamous cell	1.36	(0.73 – 2.55)	
Non-small cell§	1.04	(0.53 – 2.07)	
Large cell	16.6	(1.81 – 152.37)	
**Tumor burden**			0.113
Low tumor burden	Ref.	-	
High tumor burden††	1.50	(0.91 – 2.49)	
**Method of detection for metastatic disease****			0.015
CT surveillance§§	Ref.	-	
Symptom-based	0.34*	(0.17 – 0.71)	
Incidental	0.32*	(0.12 – 0.91)	
**Metastasis to the pleura**			0.013
Yes	Ref.	-	
No	1.72	(1.12 – 2.63)	
**Metastasis to the bone**			0.031
Yes	Ref.	-	
No	1.54	(1.04 – 2.27)	

Abbreviations. LC, Lung cancer; NOS, Not otherwise specified; LDCT, Low Dose Computed Tomography.

Note: Single imputation (Supplementary Methods 3) was performed for the Stanford Healthcare cohort prior to analysis due to a missing rate of 2.2% to 23.3% (Supplementary Table S1).

* Symptom-based and incidental detection shows a protective effect in this model due to high correlation with the exposure (de novo vs. distant recurrent metastasis).

† De novo Stage IV is defined as patients who were initially diagnosed at Stage IV with metastatic lung cancer; Distant recurrent is defined as patients who developed distant metastatic disease after an initial diagnosis of Stage I-III lung cancer.

‡ Classification of 'other' race include American Indian or Alaskan Native, Native Hawaiian or Other Pacific Islander, More than one race.

§ Classification of 'Non-small cell' include all histology types other than adenocarcinoma, squamous cell carcinoma, and large cell carcinoma.

†† High tumor burden is defined as those with tumor burden above the 75th percentile (i.e., 141.75mm).

© 2023 Su CC et al. *JAMA Network Open.*

** Method of detection was defined as surveillance if detected on a CT scan for routine follow-up or for assessing treatment response, incidental if detected on a CT scan not indicated for cancer work-up, and symptom-based if detected on interval CT scan primarily based on symptomatic complains.
§§ CT surveillance is defined as post-treatment surveillance using CT imaging (routine follow-up or assessment for treatment response) for patients who eventually developed distant recurrence and as screening using low-dose CT imaging for patients who eventually developed de novo Stage IV disease.

© 2023 Su CC et al. *JAMA Network Open.*
