# Supplementary material for: Overall Survival Among Patients With De Novo Stage IV Metastatic and Distant Metastatic Recurrent Non–Small Cell Lung Cancer
Source: JAMA Netw Open. 2023 Sep 26;6(9):e2335813. doi: 10.1001/jamanetworkopen.2023.35813 (PMC10523163; doi:10.1001/jamanetworkopen.2023.35813)
Supplement: Supplement 2. — Data Sharing Statement [file jamanetwopen-e2335813-s002.pdf]

Data Sharing Statement

Su. Overall Survival Among Patients With de Novo Stage IV Metastatic and Distant Metastatic Recurrent Non–Small Cell Lung Cancer. *JAMA Netw Open*. Published September 26, 2023. doi:10.1001/jamanetworkopen.2023.35813

Data
Data available: No

Additional Information
Explanation for why data not available: Data from the National Lung Screening Trial that support the findings of this study are made available from National Cancer Institute. Data from Stanford Healthcare are not publicly available to protect the privacy of the patients involved.
